# Glucose metabolic reprogramming: mechanisms and therapeutic implications in neuroinflammation

**DOI:** 10.3389/fimmu.2026.1737175

**Published:** 2026-06-16

**Authors:** Lan Zhang, Xinyue Yang, Xiaolin Ai

**Affiliations:** Department of Critical Care Medicine, West China Hospital, Sichuan University, Chengdu, China

**Keywords:** glycolysis, metabolic reprogramming, microglia, neurodegenerative diseases, neuroinflammation

## Abstract

Microglia play dual and context-dependent roles in the central nervous system, contributing both to the maintenance of brain homeostasis and the propagation of neuroinflammatory responses. Under pathological conditions, microglia undergo profound glycolytic reprogramming, characterized by a shift from oxidative phosphorylation to enhanced aerobic glycolysis. This review focuses on the glucose-glycolysis-lactate metabolic axis and its pivotal role in microglial immunometabolism. We elucidated how key glycolytic enzymes (e.g., HK2, PKM2) and metabolites (e.g., lactate, pyruvate, ATP) regulate microglial function through both metabolic and non-metabolic mechanisms. Furthermore, therapeutic strategies that target this glycolytic shift to alleviate neuroinflammation were discussed. A deeper understanding of microglial glycolytic reprogramming may provide critical insights for developing novel therapies for neurodegenerative diseases.

## 1 Introduction

Neuroinflammation is broadly defined as a coordinated pathological process involving microglial activation, enhanced release of proinflammatory cytokines, infiltration of peripheral immune cells, and disruption of the blood–brain barrier (BBB) (1). The neuroinflammatory hypothesis was first proposed decades ago after detecting inflammatory mediators in the postmortem brains of patients with Alzheimer’s disease (AD) and Parkinson’s disease (PD). Initially regarded as a secondary response to neuronal injury, numerous studies have suggested that neuroinflammation actively drives disease pathogenesis rather than merely reflecting it (2). Notably, the manifestations of neuroinflammation differ markedly across neurological disorders. In multiple sclerosis (MS), the pathology is mainly characterized by massive infiltration of peripheral immune cells into the central nervous system (CNS), extensive BBB breakdown, and robust glial activation. Conversely, in conditions such as AD, PD, and traumatic brain injury (TBI), neuroinflammation is characterized primarily by the activation of resident glial populations—microglia and astrocytes—and by modest elevations in inflammatory mediators (3–5).

As the principal resident immune cells of the CNS, microglia play a pivotal role in maintaining neural homeostasis. Under physiological conditions, microglia actively survey the brain parenchyma through dynamic process motility, mediate synaptic pruning, and engage in bidirectional communication with neurons, astrocytes, and oligodendrocytes to sustain network stability (6). Microglia can undergo phenotypic polarization toward a proinflammatory state under pathological stimuli (7–9). This transformation from “guardians of homeostasis” to “propagators of inflammation” underscores the dualistic nature of microglia in CNS pathology (10).

In recent years, increasing attention has been directed toward the link between microglial metabolic reprogramming and functional phenotype (11). During neuroinflammatory responses, microglia undergo a metabolic shift from oxidative phosphorylation (OXPHOS)–dominated energy production toward a glycolysis-centered metabolic profile (11). This switch facilitates rapid ATP generation and the supply of biosynthetic precursors and exerts regulatory effects on phenotypic polarization and inflammatory signaling by modulating key enzymes and intermediates in glucose metabolism (12, 13). Although several reviews have recently explored microglial metabolic reprogramming in neuroinflammation, they largely focus on broad metabolic shifts or specific pathways (14, 15). These studies provide integrated, metabolite-centric perspective that bridges glycolytic reprogramming with non-metabolic signaling and epigenetic regulation. In this review, we specifically emphasize the dual metabolic and non-metabolic roles of key enzymes (e.g., HK2, PKM2) and the emerging significance of lactate as a pleiotropic immunometabolic regulator—spanning transport, receptor signaling, and histone lactylation—in shaping microglial phenotype. Furthermore, we synthesize these mechanisms into a coherent ‘metabolic-immune-epigenetic’ axis and critically evaluate the translational challenges and disease-specific variations of targeting this axis, thereby offering a unique and clinically relevant perspective not comprehensively covered in previous reviews.

## 2 Microglia and neuroinflammation

Microglia are the resident innate immune cells of the CNS, and originate from erythromyeloid progenitors in the embryonic yolk sac (3). Studies in murine models have demonstrated that disruption of embryonic blood circulation markedly reduces microglial colonization in the developing brain, underscoring the importance of vascular integrity for their migration and distribution within the CNS. However, the precise molecular mechanisms governing microglial migration into the CNS and their subsequent diffusion throughout the brain parenchyma remain incompletely understood (3).

Upon colonizing the brain parenchyma, microglia undergo profound morphological and functional adaptations, enabling them to sense and respond to local environmental cues. Under homeostatic conditions, microglia express a signature set of markers including TMEM119, CX3CR1, Iba1, and CD11b. Among these, CX3CR1, Iba1, and CD11b are shared with peripheral macrophages and are upregulated upon microglial activation (16). CX3CR1 is particularly critical for microglia-neuron communication, mediating neuroprotective signaling and maintaining microglial surveillance. Through the CX3CR1-CX3CL1 signaling axis, microglia engage in bidirectional crosstalk with neurons, allowing them to monitor synaptic activity, remove apoptotic cells, and respond to neuronal stress (17, 18). In contrast, TMEM119 serves as a microglia-specific marker, distinguishing resident microglia from infiltrating macrophages (19). In fact, TMEM119 is not merely a marker but a functional regulator of microglial phenotype. TMEM119 deficiency disrupts the homeostatic state of microglia, accelerating their transition to the disease-associated microglia (DAM) phenotype and thereby exacerbating neuroinflammation and disease progression. Conversely, TMEM119 overexpression enhances microglial phagocytic capacity and promotes the clearance of amyloid-β (Aβ) plaques, highlighting its pivotal role in maintaining microglial homeostasis and neuroprotection (20).

Neuroinflammation broadly refers to an inflammatory response within the CNS characterized by the activation of resident immune cells, release of cytokines, and alteration of glial function (21). This process involves a complex interplay between pro-inflammatory and anti-inflammatory cytokines that collectively regulate the balance between neuroprotection and neurotoxicity (12). Among the innate immune components, microglia are the principal effectors. Microglia thus exhibit a dualistic role in neuroinflammation. Under normal or transiently injurious conditions, they exert neuroprotective effects by clearing cellular debris, apoptotic neurons, and pathological protein aggregates through phagocytosis, thereby preserving CNS homeostasis (22). Following neuronal injury, microglia can also secrete anti-inflammatory cytokines such as interleukin-10 (IL-10) and transforming growth factor-β (TGF-β) to promote tissue repair, support neuronal regeneration, and restore neural network function. However, chronic or uncontrolled microglial activation leads to sustained neuroinflammation, further aggravating neuronal loss and accelerating the progression of neurodegenerative diseases. On the one hand, activated microglia release pro-inflammatory mediators such as TNF-α, IL-1β, IL-6, and IL-8 via signaling cascades including the nuclear factor-κB (NF-κB) and mitogen-activated protein kinase (MAPK) pathways (13, 23, 24). On the other hand, persistently overactivated microglia may also cause aberrant synaptic pruning, resulting in disrupted neural connectivity and cognitive impairment (25).

## 3 Glucose metabolism and glycolysis

**Figure 1:**
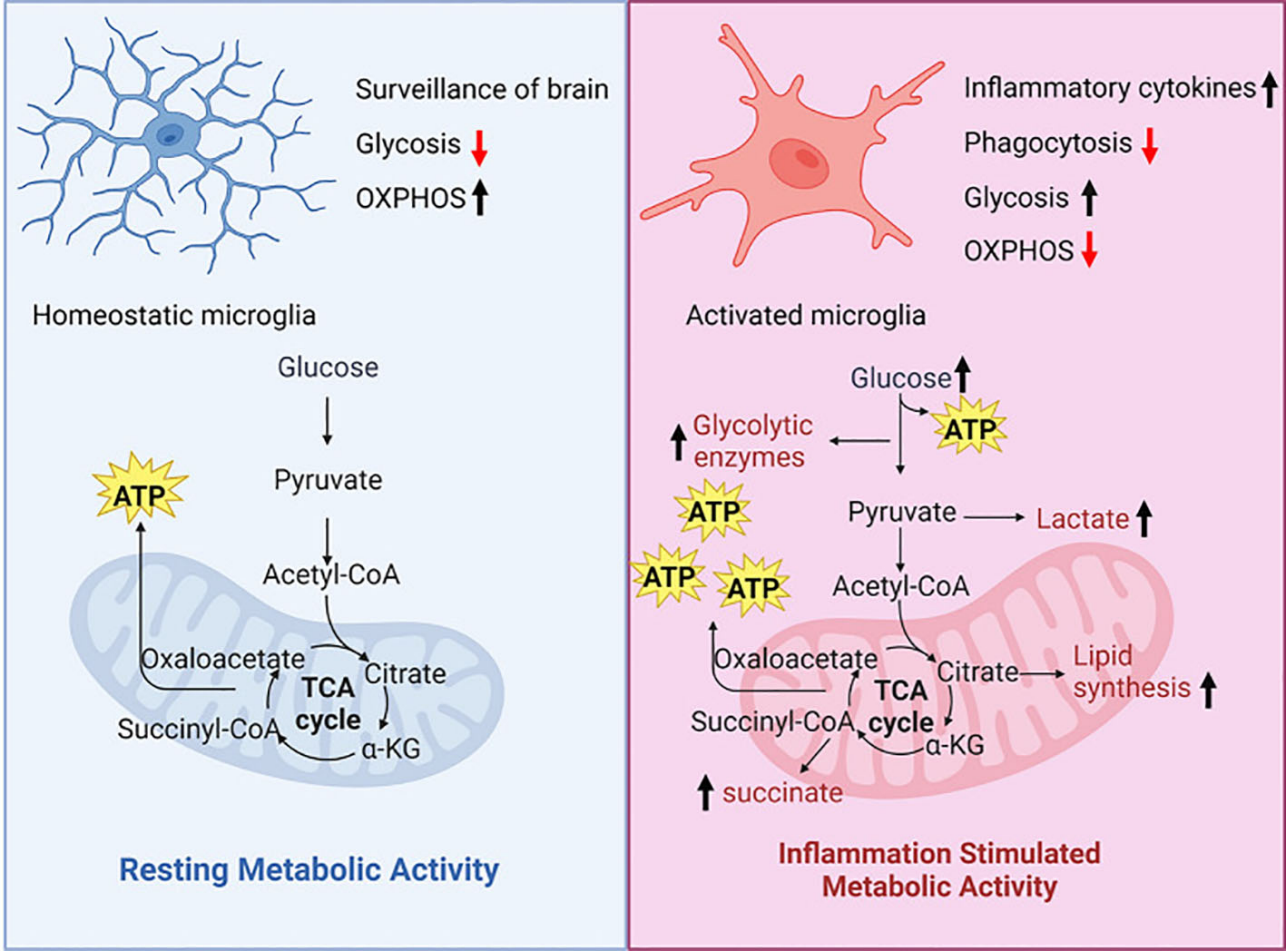
Metabolic reprogramming in microglia during phenotypic transition. The schematic illustrates the shift in microglial energy metabolism from a homeostatic to a pro-inflammatory state. Under physiological conditions (left panel), homeostatic microglia primarily rely on oxidative phosphorylation (OXPHOS) within mitochondria to generate ATP, supporting surveillance and maintenance functions. Key features include intact mitochondria with active electron transport chain (ETC) complexes and a functional tricarboxylic acid (TCA) cycle. Upon activation by pathological stimuli such as amyloid-β (right panel), microglia undergo metabolic reprogramming toward aerobic glycolysis (the Warburg effect). This shift is characterized by: (1) increased glucose uptake and glycolytic flux, (2) mitochondrial adaptation, including altered membrane potential and reduced OXPHOS efficiency, (3) accumulation of glycolytic intermediates (e.g., lactate), and (4) the involvement of key regulatory enzymes (e.g., HK2, PKM2) that bridge metabolism and inflammatory signaling. This metabolic reprogramming provides rapid ATP and biosynthetic precursors to fuel the pro-inflammatory phenotype. Created with BioRender.com.

Glucose serves as the main source of energy in the CNS, which consumes nearly 20% of that found in the blood. Under physiological conditions, glucose is metabolized to pyruvate through glycolysis in the cytosol. Pyruvate is subsequently converted into acetyl-CoA by the pyruvate dehydrogenase complex and enters the tricarboxylic acid (TCA) cycle within mitochondria. The TCA cycle generates reducing equivalents in the form of NADH and FADH_2_, which fuel the electron transport chain to drive oxidative phosphorylation (OXPHOS) and efficient ATP production. In parallel, mitochondrial respiration inevitably generates low levels of reactive oxygen species (ROS), which are tightly controlled and participate in redox signaling under homeostatic conditions (26, 27). Under homeostatic conditions, microglia primarily depend on OXPHOS for energy production, while glycolysis concurrently provides essential intermediates for biosynthetic and signaling pathways (28). This metabolic balance allows microglia to maintain a quiescent or low-activation state, ensuring readiness to respond to environmental cues without triggering excessive inflammation (29). However, under pathological or inflammatory stimulation, microglia undergo profound metabolic reprogramming, characterized by a shift from mitochondrial respiration toward aerobic glycolysis—a phenomenon analogous to the Warburg effect observed in cancer and activated immune cells (30). In this state, glucose is preferentially converted to lactate despite sufficient oxygen availability. Moreover, enhanced glycolytic flux is accompanied by functional disruption in TCA, leading to diversion of metabolites toward the pentose phosphate pathway (PPP) (31). The increased NADPH supply from PPP availability promotes ROS and nitric oxide (NO) production, forming a self-amplifying PPP-ROS-NO circuit tightly coupled with NF-κB and HIF-1α signaling (26, 32). These pathways integrate metabolic and immune signals, orchestrating transcriptional and enzymatic programs that promote the pro-inflammatory phenotype of activated microglia (**Figure 1**).

However, substantial evidence indicate that this pro-inflammatory metabolic program is reversible. Microglia can transition toward a metabolic state dominated by mitochondrial oxidative metabolism upon exposure to anti-inflammatory factor (e.g. IL-4,IL-13) (33). This state is characterized by preserved oxygen consumption rates, sustained ATP generation and a relatively reduced glycolytic flux. Concurrently, the PPP is attenuated. At the level of molecular regulation, AMP-activated protein kinase (AMPK) and IL-10–dependent signaling pathways are thought to play central roles by suppressing mTOR activity, restraining glycolytic induction, and supporting mitochondrial integrity and function (34). Collectively, this metabolic–signaling axis supports long-term functions associated with tissue repair, debris clearance, and the resolution of inflammation.

## 4 Key enzymes and metabolites in microglial glucose metabolic reprogramming

### 4.1 Hexokinase 2

Hexokinase 2 (HK2) catalyzes the first and rate-limiting step of glycolysis, phosphorylating glucose to generate glucose-6-phosphate (G6P). Accumulating evidence suggests that HK2 is selectively and highly expressed in microglia in the murine brain and its expression is markedly upregulated in microglia under immune stimulation (35). Genetic ablation of HK2 significantly reduces glycolytic flux and ATP production in microglia, accompanied by impaired homeostatic surveillance and diminished directed migration (36). However, complete HK2 loss does not necessarily lead to neuroinflammation resolution. In mouse models of ischemic stroke, HK2 deficiency paradoxically exacerbates neuroinflammation and enlarges tissue injury by inducing mitochondrial dysfunction and excessive ROS accumulation (37). Notably, HK2 has been reported to contribute to mitochondrial homeostasis by binding to the voltage-dependent anion channel (VDAC) on the outer membrane, thereby stabilizing mitochondrial membrane potential, limiting excessive ROS release, and restraining NLRP3 inflammasome activation (38). Consistent with this notion, studies indicate that complete HK2 ablation does not ameliorate pathology and may instead exacerbate disease progression, whereas HK2 haploinsufficiency reduces amyloid-β burden in 5xFAD mice (39). Collectively, these findings suggest that HK2 functions as both a glycolytic driver and an immunometabolic regulatory node in microglia, with its impact on neuroinflammation appearing to be dose-dependent rather than uniformly beneficial or detrimental (39).

### 4.2 Pyruvate kinase M2

Pyruvate kinase M2 (PKM2), a key rate-limiting enzyme in the terminal step of glycolysis, catalyzes the conversion of phosphoenolpyruvate (PEP) to pyruvate. PKM2 exists in two distinct conformational states. The tetrameric form of PKM2 exhibits high catalytic activity and primarily supports glycolytic flux and ATP production, whereas the dimeric form favors metabolic rewiring and pro-inflammatory transcriptional programs. The dimeric form can translocate into the nucleus, where it acts as a transcriptional co-regulator for factors such as hypoxia-inducible factor-1 (40, 41). In response to inflammatory stimuli, PKM2 expression is markedly upregulated in microglia and has been implicated in the amplification of pro-inflammatory responses (34). Although PKM2 is a glycolytic enzyme, accumulating evidence indicates that its pro-inflammatory role in microglia is largely independent of its contribution to glycolytic flux. Instead, the dimeric and nuclear form of PKM2 functions as a metabolic signaling molecule that directly regulates inflammatory gene transcription. For example, during the acute phase of traumatic brain injury(TBI) in mice, treatment with TEPP-46, a pharmacological inhibitor of PKM2 dimerization, significantly attenuates microglia-mediated inflammatory responses, improves mitochondrial function and behavioral performance (34). Consistently, exposure to triclosan (TCS) has been shown to robustly activate microglia in the prefrontal cortex, and treatment with TEPP-46, effectively blocks TCS-induced inflammatory gene expression (42). Therefore, compared with global inhibition of PKM2, accumulating evidence indicates that, in the context of neurodegenerative diseases, stabilizing PKM2 in its tetrameric conformation and restricting its dimerization and nuclear translocation allows for selective suppression of inflammatory responses without markedly disrupting basal metabolic functions (34). Collectively, these findings highlight PKM2 as a crucial metabolic and signaling hub that integrates energy metabolism with transcriptional control of neuroinflammatory responses.

### 4.3 Lactate

**Figure 2:**
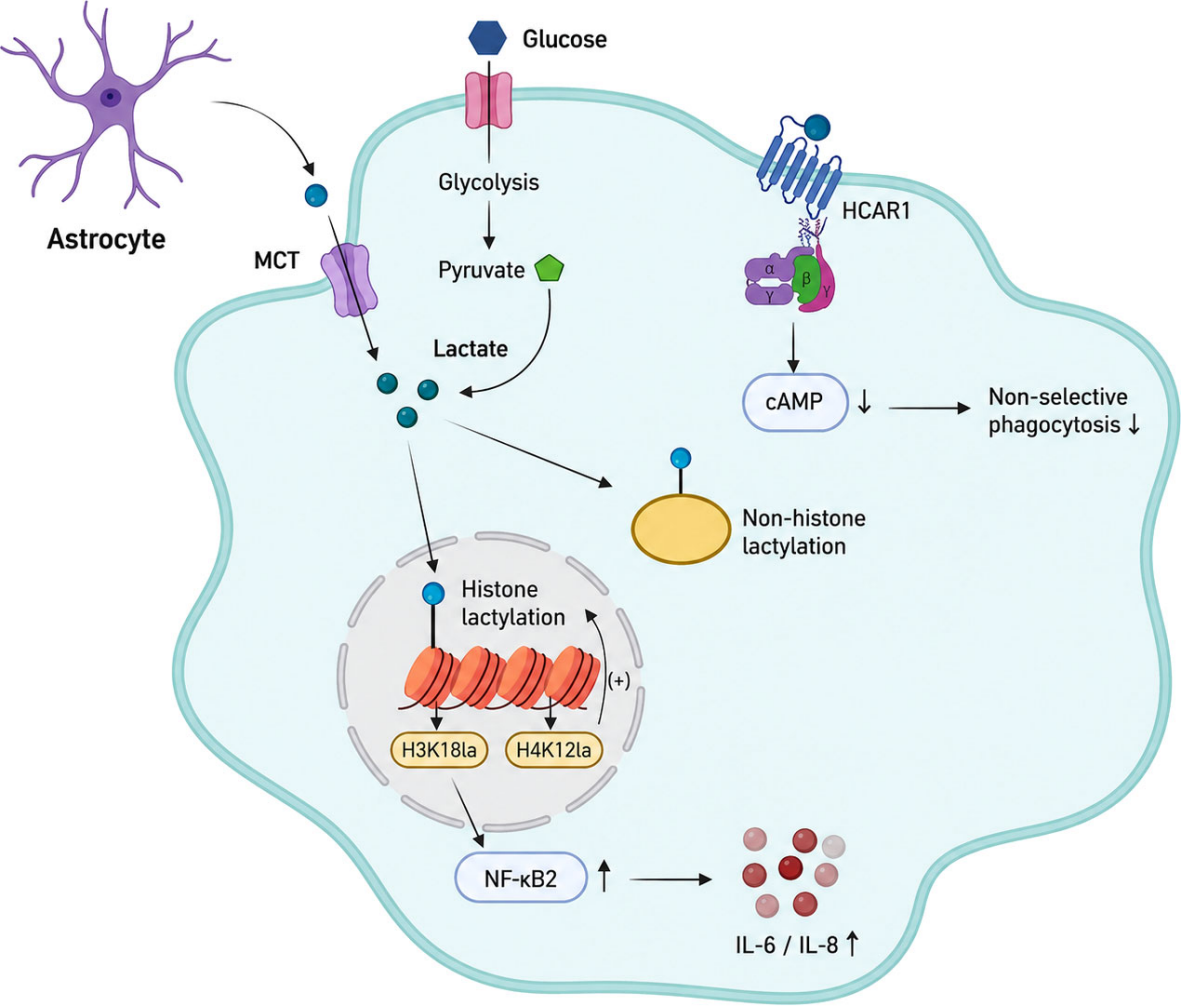
Multifaceted mechanisms of lactate in regulating microglial function. The figure details specific pathways through which lactate acts as a signaling molecule and epigenetic modifier. The diagram comprises several interconnected panels: 1. Transport (Top Left): Monocarboxylate transporters (MCT1 and MCT4) facilitate the bidirectional shuttling of lactate across the microglial plasma membrane, influenced by intra- and extracellular concentration gradients; 2. Receptor Signaling (Top Right): Extracellular lactate activates the hydroxycarboxylic acid receptor 1 (HCAR1/GPR81), a Gi-protein coupled receptor. This engagement inhibits adenylate cyclase (AC), reduces intracellular cyclic AMP (cAMP) levels, and suppresses protein kinase A (PKA) activity, leading to attenuated pro-inflammatory responses; 3. Epigenetic Regulation (Histone Lactylation, Bottom Left): Intracellular lactate serves as a substrate for histone lactylation, a novel post-translational modification. The enzyme catalyzing this transfer is implied. Lactylation on histone tails (e.g., H3K18, H4K12) alters chromatin structure and promotes the transcription of specific gene sets, which can be either pro-repair or pro-inflammatory depending on context; and 4. Non-histone Protein Lactylation (Bottom Right): Lactylation also modifies non-histone proteins, affecting their activity, stability, or interactions. This widespread modification impacts core cellular processes including glycolysis, cytoskeletal dynamics, and inflammatory signaling pathways, thereby integrating metabolic state with functional output. Collectively, these panels illustrate how lactate transcends its metabolic role to become a central regulator of microglial immunometabolism through transport, receptor-mediated signaling and epigenetic proteomic reprogramming. Created with BioRender.com.

Lactate is not merely a metabolic byproduct of glycolysis but a pleiotropic immunoregulatory metabolite that modulates microglial functional states through multiple mechanisms, including transporter-mediated regulation, receptor signaling, epigenetic modification, and protein functional reprogramming (43, 44) (**Figure 2**). Microglia predominantly express monocarboxylate transporters (MCTs), particularly MCT1 and MCT4, which mediate bidirectional lactate transport according to the transmembrane concentration gradient (45, 46). Under homeostatic conditions, MCT1 primarily facilitates the uptake of extracellular lactate, whereas during pro-inflammatory activation, MCT4 promotes lactate efflux from the cytosol. Pharmacological inhibition of MCT1 markedly suppresses LPS-induced glycolytic upregulation and the subsequent release of inflammatory cytokines. Conversely, MCT4 upregulation supports the export of excess intracellular lactate to maintain cytosolic pH balance and metabolic homeostasis (45). Loss of MCT4 disrupts lactate-driven lysosomal acidification, thereby impairing microglial synaptic pruning (47). Although MCTs are not classical signaling receptors, their regulation of lactate flux critically influences microglial activation states, making them essential components of the lactate-mediated immunometabolic network.

Beyond transport mechanisms, lactate also functions as a signaling molecule through the hydroxycarboxylic acid receptor (HCAR) family, a subset of G protein–coupled receptors (GPCRs). Among these, HCAR1 (also known as GPR81) is inducibly upregulated in microglia under pathological stress, such as elevated extracellular lactate levels, ischemia, or traumatic brain injury (48). Upon activation by lactate, HCAR1 engages the Gi–cAMP signaling cascade, which exerts a negative feedback effect by dampening excessive immune activation and limiting non-selective phagocytosis (49, 50). This receptor-mediated pathway exemplifies lactate’s capacity to act as a metabolic checkpoint, preventing overactivation of microglial inflammatory responses.

In addition to its signaling roles, accumulating evidence indicates that microglia undergo metabolic reprogramming under pathological conditions, leading to lactate accumulation and a marked increase in histone lactylation. In Alzheimer’s disease models, H4K12 lactylation (H4K12la) activates PKM2 expression, forming a glycolysis–H4K12la–PKM2 positive feedback loop that exacerbates microglial activation and amyloid-β pathology (51). In parallel, H3K18 lactylation (H3K18la) enhances binding at the NF-κB1 promoter, upregulates senescence-associated secretory phenotype (SASP) factors such as IL-6 and IL-8, and drives microglial senescence and chronic inflammation (52). In spinal cord injury models, ischemia- and hypoxia-induced lactate elevation enhances H4K12la, thereby promoting microglial scar formation and improving motor function recovery (53). In addition, physical exercise increases brain lactate levels, induces beneficial histone lactylation, suppresses excessive microglial activation, and promotes the expression of repair-associated genes (54).

Although specific lactylation sites and functional consequences vary across studies (55, 56), histone lactylation is not merely a pathogenic modification, but rather a highly plastic and context-dependent epigenetic regulatory mechanism. Targeting the metabolism–lactate–histone lactylation–microglial function axis may therefore offer novel therapeutic opportunities for neurodegenerative diseases and brain injury.

Recent advances in proteomics further reveal that non-histone lactylation is widespread, modifying proteins involved in cellular metabolism, cytoskeletal organization, and signal transduction (57, 58). Collectively, these findings establish lactate as a central signaling metabolite that orchestrates microglial metabolism, inflammatory signaling, and gene expression across multiple regulatory layers, positioning it as a pivotal mediator of neuroimmune homeostasis and dysfunction.

### 4.4 ATP and purinergic signaling

Adenosine triphosphate (ATP), classically known as the universal “energy currency” of the cell, also functions as a damage-associated molecular pattern (DAMP) within the central nervous system (CNS), capable of initiating and amplifying neuroinflammatory responses (59, 60). Notably, the production and release of ATP are highly dependent on the cellular glycolytic state, rendering ATP not only a reflection of energy metabolism but also a key mediator through which metabolic reprogramming is translated into immune signaling. Through purinergic signaling, extracellular nucleotides such as ATP and its metabolites act as context-dependent modulators of microglial activation, orchestrating the dynamic balance between pro-inflammatory and neuroprotective phenotypes.

Early studies on microglial purinergic signaling primarily focused on ionotropic P_2_X receptors and metabotropic P_2_Y receptors, which mediate distinct functional outcomes depending on pathological context. Under conditions such as cerebral ischemia and Alzheimer’s disease (AD), inflammation-associated stimuli are typically accompanied by enhanced glycolytic flux in microglia, concomitant with a marked upregulation of P2X receptors, particularly P2X7 and P2X4 (61, 62). Activation of these receptors promotes microglial process extension, migration, and release of pro-inflammatory cytokines, while triggering NLRP3 inflammasome activation, thereby establishing a self-sustaining inflammatory feedback loop that exacerbates neurodegeneration. In contrast, P_2_Y receptors, including P_2_Y_12_ and P_2_Y_6_, are predominantly expressed in homeostatic microglia, a state that is generally associated with lower glycolytic activity and a greater reliance on oxidative metabolism, where they regulate phagocytic initiation, chemotactic process motility, and maintenance of synaptic integrity (63–65). Thus, P_2_X- and P_2_Y-mediated signaling represent complementary yet opposing regulatory axes in microglial immunometabolism.

Recent research attention has increasingly shifted toward downstream purine metabolites, particularly adenosine, which acts through four G protein–coupled receptors—A_1_R, A_2_AR, A_2_BR, and A_3_R—all expressed on microglia. Compared with ATP, adenosine signaling often becomes more prominent under sustained glycolytic and inflammatory conditions and is thought to participate, at least in part, in constraining excessive amplification of metabolic–inflammatory cascades (66). Among these, A_1_R has been widely recognized for its neuroprotective effects, including inhibition of adenylyl cyclase, suppression of intracellular cAMP accumulation and calcium influx, attenuation of glutamate release, and facilitation of CX3CL1-mediated neuron–microglia communication (67, 68). A_3_R, by contrast, has emerged as a promising analgesic target, as its activation on perineuronal macrophages and spinal microglia alleviates neuropathic pain and dampens neuroinflammation (69).

Nevertheless, the role of A_2_AR remains context-dependent and mechanistically complex. Although A_2_AR activation confers neuroprotection in ischemic brain injury through cAMP-dependent anti-inflammatory pathways, others have demonstrated that excessive or prolonged A_2_AR signaling may potentiate microglial activation and exacerbate neuroinflammatory damage in chronic neurodegenerative conditions (66, 70). This bidirectional behavior underscores the dual nature of adenosine signaling and functionings as both a homeostatic regulator and a pathological amplifier depending on microenvironmental cues.

### 4.5 Pyruvate and ethyl pyruvate

Pyruvate serves as a central metabolic hub connecting glycolysis to the TCA cycle, thereby playing a pivotal role in integrating cellular energy metabolism with redox homeostasis and inflammatory signaling (71, 72). Beyond its canonical function as a metabolic intermediate, pyruvate modulates the NAD^+^/NADH ratio, reactive oxygen species (ROS) production, and mitochondrial activity, thereby actively participating in glycolysis-driven immunometabolic reprogramming in microglia (73). Under conditions of glucose restriction or impaired mitochondrial oxidative metabolism, microglia exhibit an increased reliance on glycolysis. In this context, pyruvate sustains its intracellular and transmembrane flux through upregulation of monocarboxylate transporter 1 (MCT1) and activates the NF-κB signaling pathway, thereby supporting the persistence of a pro-inflammatory phenotype and amplifying cytokine release and inflammatory responses. Collectively, these findings indicate that pyruvate is not merely the terminal product of glycolysis, but rather a key signaling node that translates glycolytic status into inflammatory phenotypic outcomes.

Within this metabolic framework, nicotinamide adenine dinucleotide (NAD^+^) functions as both a redox cofactor and a signaling metabolite that couples glycolytic activity to mitochondrial quality control and inflammatory regulation (74). Elevated NAD^+^ availability favors activation of the NAD^+^-dependent deacetylase Sirtuin 1 (SIRT1), which attenuates microglial inflammatory signaling through deacetylation and suppression of NF-κB subunits (75, 76). Concurrently, SIRT1 engages the PGC-1α–PINK1/Parkin axis to promote mitophagy, thereby preserving mitochondrial integrity, limiting ROS accumulation, and restraining chronic microglial activation. In this context, the glycolysis–pyruvate–NAD^+^ axis emerges as an integrated immunometabolic circuit coordinating energy metabolism, redox homeostasis, and inflammatory tone in microglia (77). Conversely, under sustained inflammatory stress, excessive NAD^+^ consumption by enzymes such as CD38 and poly(ADP-ribose) polymerase-1 (PARP1) disrupts this metabolic equilibrium. Depletion of intracellular NAD^+^ compromises mitochondrial metabolism, impairs phagocytic capacity, and predisposes microglia to bioenergetic failure and necroptotic cell death, thereby reinforcing neuroinflammatory cascades (78–80).

However, the chemical instability of pyruvate in aqueous environments—where it readily undergoes degradation and polymerization—poses significant challenges for its experimental reproducibility and clinical application. To overcome these limitations, researchers have turned to ethyl pyruvate (EP), an esterified and more stable analog of pyruvate that exhibits enhanced lipophilicity and blood–brain barrier permeability (71). Although EP cannot be enzymatically converted back into pyruvate *in vivo*, it retains and even extends pyruvate’s biological activities, particularly in the context of neuroinflammation and microglial immunometabolism.

EP has demonstrated potent anti-inflammatory and neuroprotective effects, primarily through suppression of microglial activation and inhibition of the NLRP3 inflammasome, resulting in reduced secretion of pro-inflammatory cytokines (81–83). Mechanistically, EP modulates the NF-κB/High Mobility Group Box 1 (HMGB1) signaling axis-a key pathway in sterile inflammation. HMGB1, once released extracellularly as a damage-associated molecular pattern (DAMP), interacts with Toll-like receptors (TLRs) and the Receptor for Advanced Glycation End-products (RAGE) to amplify neuroinflammatory cascades. EP disrupts this amplification loop by chelating intracellular Ca²^+^ and inhibiting HMGB1 phosphorylation, thereby preventing its nuclear export and subsequent extracellular release (82, 84). Therefore, these findings position EP as a promising pharmacological modulator of microglial metabolism and inflammatory signaling. By targeting the metabolic-inflammatory interface, EP represents a potential therapeutic approach for mitigating metabolic dysregulation-driven neuroinflammation in disorders such as Alzheimer’s disease, ischemic stroke, and traumatic brain injury.

## 5 Integrated mechanisms of glycolytic reprogramming in microglia

**Figure 3:**
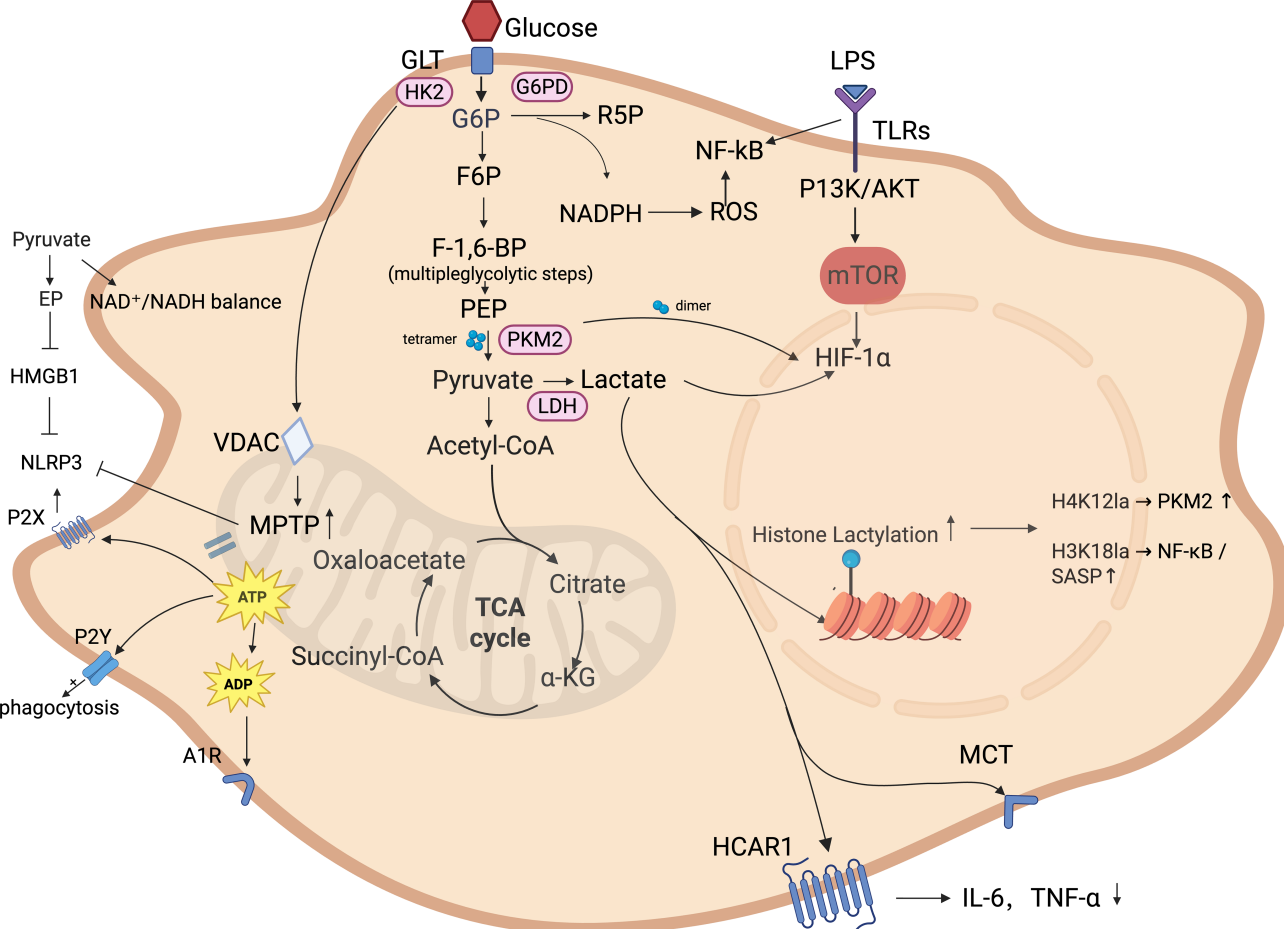
Glucose metabolic reprogramming and immunometabolic regulation in microglia. Enhanced glucose uptake and glycolytic flux drive metabolic branching toward lactate production and mitochondrial metabolism. Glucose-6-phosphate (G6P) is diverted into the pentose phosphate pathway (PPP) to generate NADPH, which context-dependently regulates redox balance and inflammatory signaling. Pyruvate kinase M2 (PKM2) acts as a metabolic checkpoint, where its tetrameric form supports energy homeostasis, whereas dimerization and nuclear translocation promote HIF-1α–dependent transcriptional programs. Pyruvate is reduced to lactate by lactate dehydrogenase (LDH), facilitating NAD^+^ regeneration and enabling sustained inflammatory glycolysis. Lactate functions as a signaling metabolite by inducing histone lactylation, including H4K12la and H3K18la, thereby reinforcing PKM2 expression, NF-κB–dependent transcription, and senescence-associated secretory phenotypes. In parallel, ATP release and purinergic signaling through P2X and P2Y receptors regulate microglial activation and phagocytic behavior. Ethyl pyruvate (EP) pharmacologically attenuates neuroinflammation by suppressing HMGB1 release, NLRP3 inflammasome activation, and mitochondrial dysfunction. Created with BioRender.com.

Collectively, these findings illustrate that glycolytic reprogramming in microglia is not merely a metabolic adaptation for energy supply, but a tightly coordinated regulatory program that translates inflammatory cues into sustained immune activation. Pathological stimuli, including LPS, amyloid-β (Aβ), damage-associated molecular patterns (DAMPs), and extracellular ATP, activate central signaling axes such as mTOR–HIF-1α and NF-κB, thereby initiating glycolysis-centered metabolic remodeling (2).(**Figure 3**).

## 6 Disease-specific metabolic reprogramming of microglia across neurological disorders

Neuroinflammation is convergent pathological feature across a broad spectrum of neurological disorders. In this inflammatory setting, microglia exhibit distinct metabolic states and reprogramming profiles that depend on disease-specific stimuli, temporal progression, and immune environments. In this section, microglia metabolic reprogramming is illustrated across NDDs.

Glycolytic reprogramming has emerged as a common metabolic feature of microglial activation in Alzheimer’s disease (AD), Parkinson’s disease (PD), and multiple sclerosis (MS), reflecting a shared shift from oxidative phosphorylation toward aerobic glycolysis in microglial metabolism under inflammatory conditions across these disorders (21, 24). This switch is consistently associated with increased lactate production, altered NAD^+^/NADH redox balance, and induction of pro-inflammatory transcriptional programs. However, the molecular triggers and initial metabolic constraints driving this glycolytic shift exhibit certain differences among these diseases. In the AD mouse model, the accumulation of β-amyloid, early mitochondrial dysfunction, and the release of mitochondrial DNA as damage-associated molecular patterns collectively impose metabolic stress on microglia, promoting an initial glycolytic response that precedes and amplifies neuroinflammation (44, 85, 86). In the PD model, misfolded α-synuclein acts as the primary upstream signal, forcing glycolytic reprogramming, with research indicating sustained activation of the AKT-mTOR-HIF-1α axis and PKM2-dependent metabolic regulation, thereby linking pathogenic protein aggregation with inflammatory metabolism (87, 88). In MS, glycolytic activation in microglia is not driven by a singular intrinsic neurodegenerative trigger but occurs within a highly inflammatory lesion microenvironment shaped by blood–brain barrier disruption and infiltration of peripheral immune cells (89, 90). Thus, while glycolysis represents a common metabolic endpoint of microglial activation, its induction reflects distinct pathological pressures imposed by neurodegeneration versus immune-mediated tissue damage.

Beyond differences in initiation and constraints, the temporal persistence and metabolic resilience of glycolytic reprogramming are key factors distinguishing microglial responses in NDDs, directly influencing disease progression. In AD, growing evidence supports a stage-dependent metabolic trajectory, where early glycolytic activation transitions to late-stage metabolic insufficiency characterized by reduced glycolytic flux, impaired mitochondrial function, and diminished immune capacity (86, 91). This loss of metabolic flexibility coincides with chronic inflammasome activation, epigenetic reinforcement of inflammatory states through lactate-driven histone lactylation, and progressive impairment of homeostatic functions such as phagocytosis and synaptic support (7, 52). In contrast, PD is marked by prolonged, glycolysis-dominated inflammatory microglial phenotypes, sustained α-synuclein signaling, and mitochondrial electron transport chain dysfunction, which together may contribute to prolonged inflammatory responses, heightened oxidative stress, and neurotoxic microglia-neuron interactions that accelerate dopaminergic neuron loss (56, 92, 93). Meanwhile, the metabolic state of microglia in MS exhibits notable spatial and lesion-stage heterogeneity, with glycolysis playing context-dependent roles in debris clearance, lipid processing, and lesion remodeling rather than uniformly driving tissue damage (90, 94). However, direct, systematic comparative studies on these microglial metabolic alterations across neurodegenerative diseases remain scarce.

Collectively, these comparisons indicate that glycolytic reprogramming in microglia is not inherently pathogenic; rather, its contribution to neurodegeneration or tissue repair depends on temporal dynamics, metabolic resilience, and microenvironmental context.

## 7 Targeting neuroinflammation for CNS disease treatment

Targeting neuroinflammation has emerged as an important therapeutic avenue for CNS diseases. In neurodegenerative disorders, microglia, the principal innate immune effector cells of the CNS, are thought to contribute to disease progression through dysregulated inflammatory signaling, including cytokine release, complement activation, metabolic reprogramming, and immune dysfunction, thereby motivating the development of therapeutic strategies that target distinct functional aspects of microglial regulation (**Table 1**).

**Table 1:** Representative therapeutic strategies targeting neuroinflammation.

Therapeutic category	Representative approach	Therapeutic mechanism	Targeted key enzyme and metabolite pathway	References
Regulation of microglial glycolytic metabolism	Cordycepin	Suppress aberrant glycolytic flux and pro-inflammatory activation	HK2, PDK2,	(95)
	Ononin	Suppress aberrant glycolytic flux and pro-inflammatory activation	PKM2	(96)
	lncRNA-AC020978	Enhances glycolysis and inflammatory activation	PKM2 expression and phosphorylation	(97)
	Oxygen-loaded nanodroplets	Alleviate hypoxia and inhibit HIF-1α–driven glycolysis	HIF-1α signaling	(98)
	Agmatine	Inhibits glycolysis and restores mitochondrial function	PI3K/Akt/mTOR/HIF-1α axis	(99)
	Metformin; Propofol; DW14006	Suppress glycolysis-driven inflammation	AMPKα1–HIF-1α pathway	(100–102)
	Itaconate; Dimethyl fumarate	Modulate mitochondrial respiration and redox balance	TCA cycle and mitochondrial respiratory chain	(103)
	Tetrandrine	Inhibits inflammatory signaling	TLR4/NF-κB pathway	(104)
	Minocycline	Suppresses glycolysis-driven inflammatory phenotypes	EMB/MCT4/STING axis	(105)
Microglial replacement therapy	Mr BMT	Replace dysfunctional microglia	CSF1R-dependent microglial niche	(106)
	tBMT	Partial microglial replacement	Bone marrow–derived myeloid cells	(107, 108)
Cytokine inhibition strategies	NSAIDs	Reduce prostaglandin synthesis	COX enzymes	(109, 110)
	Infliximab	Neutralizes cytokine signaling	TNF-α	(111)
	Anakinra	Blocks cytokine receptor signaling	IL-1 receptor	(111)
	Tocilizumab	Inhibits cytokine-driven immune activation	IL-6 receptor	(112)
Complement system modulation	Eculizumab	Inhibits terminal complement activation	C5	(113)
	PMX205; Avacopan	Block complement-mediated inflammation	C5aR1	(114, 115)

### 7.1 Regulation of microglial glycolytic metabolism

As discussed above, glycolytic metabolism is tightly linked to microglial function, and precise regulation of microglial glycolysis has emerged as a key immunometabolic strategy for modulating neuroinflammation and the progression of neurodegenerative diseases. Cordycepin and ononin suppress aberrantly enhanced glycolytic flux in microglia by targeting HK2, pyruvate dehydrogenase kinase 2 (PDK2), and PKM2, thereby alleviating inflammatory responses (95, 96). In contrast, the long non-coding RNA lncRNA-AC020978 promotes PKM2 expression and phosphorylation, enhances glycolytic flux, and drives inflammatory activation of microglia (97). Oxygen-loaded nanodroplets alleviate local hypoxia and suppress hypoxia-driven HIF-1α activation, indirectly constraining glycolytic reprogramming and reducing microglial inflammatory activation (98). Agmatine inhibits excessive glycolytic flux through the PI3K/Akt/mTOR/HIF-1α signaling axis, improves mitochondrial function in inflammation-stimulated microglia, and attenuates pro-inflammatory phenotypes (99). In addition, metformin, propofol, and the direct AMPKα1 activator DW14006 regulate microglial glucose metabolism via the AMPK/HIF-1α pathway, suppress glycolysis-driven inflammatory responses, and improve cognitive outcomes across multiple disease models (100–102). As representative immunometabolic regulators, itaconate and its derivative dimethyl fumarate differentially modulate mitochondrial respiratory chain activity, alleviate oxidative stress associated with mitochondrial hyperactivity, and restore metabolic homeostasis in microglia (103). Moreover, the natural alkaloid tetrandrine suppresses TLR4/NF-κB–mediated pro-inflammatory signaling (104). Long-term minocycline treatment further inhibits glycolysis-driven inflammatory phenotypes of microglia by regulating the EMB/MCT4/STING axis (105).

In addition, several microglia-targeted therapies have already entered clinical evaluation. TREM2 agonists enhance microglial survival and phagocytic capacity by activating downstream signaling pathways such as PI3K–AKT/mTOR (116, 117). In parallel, Aβ-directed immunotherapeutic strategies, such as lecanemab, primarily reduce cerebral pathological burden and thereby indirectly alleviate sustained microglial activation (118).

Collectively, these studies demonstrate that modulation of glycolytic enzymes, blockade of HIF-1α–dependent metabolic programs, restoration of mitochondrial oxidative metabolism, and coordinated regulation of inflammation-related signaling pathways can systematically reshape microglial inflammatory and phagocytic functions, providing a unified immunometabolic framework for therapeutic intervention in neuroinflammation and neurodegenerative diseases.

### 7.2 Microglial replacement therapy

Microglial replacement therapy (MRT), represents a novel therapeutic strategy for neurodegenerative disorders. MRT encompasses several distinct approaches, primarily including microglial replacement via bone marrow transplantation (Mr BMT), microglial replacement via peripheral blood–derived myeloid cells (Mr PB) and direct microglial transplantation (Mr MT). Mr BMT has advanced to human clinical studies and demonstrated therapeutic efficacy (106). In ALSP mouse models, a representative white matter disease caused by CSF1R mutations (119, 120), Mr BMT effectively replaces pathogenic microglia and slows disease progression (121). In addition, prior to the development of Mr BMT, traditional bone marrow transplantation (tBMT) has shown therapeutic benefit in ALSP and other rapidly progressive neurodegenerative disorders (107, 108). These effects are thought to involve the migration of bone marrow–derived cells into the central nervous system, where they differentiate into long-lived microglia-like cells. Although tBMT, compared with Mr BMT, achieves less efficient microglial replacement within the central nervous system, both approaches achieve comparable therapeutic outcomes by replacing dysfunctional microglia and improving neuronal function in ALSP model, highlighting the translational potential of microglial replacement–based therapies (121).

### 7.3 Cytokine inhibition strategies

Elevated levels of pro-inflammatory cytokines, including interleukin-1 (IL-1), tumor necrosis factor-α (TNF-α), and interleukin-6 (IL-6), are consistently observed across neurological disorders and contribute to sustained neuroinflammation (122). Early anti-inflammatory approaches have often involved the use of nonsteroidal anti-inflammatory drugs (NSAIDs), inhibit cyclooxygenase (COX) activity and reduce prostaglandin synthesis, thereby decreasing the production of pro-inflammatory mediators. Although epidemiological studies have associated NSAID use with a reduced risk of neurodegenerative diseases and preclinical models have demonstrated their potential anti-neuroinflammatory effects, their clinical application remains limited by insufficient blood–brain barrier (BBB) penetration and systemic toxicities (109, 110). Consequently, precision therapies targeting specific inflammatory mediators have gained increasing attention. Biologic agents directed against TNF-α, IL-1, or IL-6, including infliximab, anakinra, and tocilizumab, have shown anti-inflammatory and neuroprotective effects in preclinical models and have been explored in neurological disorders (111, 112).

In parallel, medicinal plants and their bioactive compounds, including extracts from Curcuma longa, Cannabis sativa, and apigenin, have demonstrated the ability to suppress pro-inflammatory cytokine production and inhibit COX-2 and inducible nitric oxide synthase (iNOS), largely through activation of Nrf2 signaling and inhibition of NF-κB pathways (123–125).

Beyond classical cytokines, emerging evidences show inflammatory regulators such as chitinase-3-like protein 1 (CHI3L1), the protein tyrosine phosphatase Shp2, and galectin-3 have been identified as key signaling hubs that integrate immune activation, metabolic stress, and cellular injury (126–128). Through their bidirectional regulatory properties, these molecules mediate feedback regulation and signaling crosstalk among multiple inflammatory pathways, expanding the landscape of precision anti-inflammatory targets.

However, clinical translation remains limited by poor blood–brain barrier penetration, the pleiotropic nature of cytokine signaling, and safety concerns associated with long-term systemic immunosuppression (129). To overcome these barriers, novel delivery strategies, including oral lipid nanoparticles, interventions, gene- and RNA-based interventions, as well as advanced nanoparticle delivery systems, are being actively developed to improve CNS targeting and therapeutic specificity (130–132).

### 7.4 Dynamic balance of complement system

The complement system, a key component of innate immunity, plays dual roles in neuroinflammation and synaptic homeostasis. Under pathological conditions, complement activation contributes to aberrant synaptic pruning, inflammatory amplification, and neurotoxicity, as reported in Alzheimer’s disease, sepsis-associated encephalopathy, and ischemic brain injury (133–135). Conversely, complement activity is also required for physiological synaptic development and tissue repair. Accordingly, recent studies have argued against global inhibition of the complement cascade and have instead focused on downstream effector pathways, particularly the C5 or C5a–C5aR1 axis (136, 137). Interventions targeting these pathways, including eculizumab, the small-molecule C5aR1 antagonist PMX205 and Avacopan, have been widely evaluated across multiple disease settings and have shown acceptable tolerability in clinical and preclinical studies (113–115).

## 8 Future directions and challenges

Although targeting glycolytic reprogramming presents a promising avenue for modulating microglia, several key challenges and future directions must be addressed to advance the field (125, 138). First, the spatial and temporal heterogeneity of microglial metabolic states in different brain regions and across disease stages remains poorly mapped. Techniques like single-cell metabolomics and spatial transcriptomics are needed to correlate specific metabolic fluxes with functional phenotypes *in vivo*. Second, the interplay between glycolysis and other major metabolic pathways—such as lipid metabolism (fatty acid oxidation, lipid droplet dynamics), amino acid utilization, and mitochondrial TCA cycle rewiring — is crucial for a holistic understanding of microglial immunometabolism. How these pathways compensate or conflict with glycolytic shifts during neuroinflammation warrants systematic investigation. Third, the translational potential of metabolic interventions faces the dual hurdles of blood-brain barrier (BBB) penetration and cell-type specificity. Developing brain-penetrant prodrugs, nanoparticle delivery systems, or context-specific activators/inhibitors is essential. Finally, the impact of systemic metabolism on microglial function, such as the effects of ketogenic diets, fasting, or exercise on neuroinflammation through microglial metabolic remodeling, represents an exciting frontier for both mechanistic research and non-pharmacological therapeutic strategies. Addressing these questions will not only refine our understanding of microglial biology but also pave the way for precise and effective metabolic interventions against neuroinflammatory diseases (106).

## 9 Conclusion

Neuroinflammation is now recognized as a central and active driver of NDDs, with microglia acting as key regulators through dynamic changes in activation state, phenotype, and metabolic programs. Growing evidence indicates that neuroinflammatory progression is tightly coupled to microglial reprogramming of glucose metabolism, underscoring metabolic state as a critical determinant of microglial function. Therapeutic strategies targeting microglial glucometabolic reprogramming, microglial replacement therapy, as well as cytokine inhibition and complement modulation, are collectively shaping a multidimensional framework for neuroinflammation intervention and advancing our understanding of how inflammatory, metabolic, and immune signals converge to shape disease outcomes. Nevertheless, major knowledge gaps remain regarding the context-dependent integration of these signals across cellular states, spatial regions, and disease stages. Addressing these gaps will be essential for translating this emerging therapeutic paradigm into safe, controllable, and broadly applicable treatments for NDDs.
